# Informing the cultural adaptation of a lung cancer screening shared decision-making and navigation intervention for Hispanic adults: a qualitative study

**DOI:** 10.1007/s10552-026-02257-7

**Published:** 2026-09-23

**Authors:** Evelyn Arana-Chicas, Alex Varela Reyes, Arlette Chavez-Iniguez, Myneka Macenat, Jeanne Ferrante, Camila Capurro, Anita Y. Kinney

**Affiliations:** 1 Division of Medical Oncology, Rutgers Cancer Institute, 120 Albany Street, Tower 2, 8thFloor, New Brunswick, NJ 08901 USA; 2 Department of Family Medicine, Robert Wood Johnson Medical School, New Brunswick, NJ 08901 USA; 3 Department of Biostatistics and Epidemiology, Rutgers School of Public Health, New Brunswick, NJ 08854 USA

**Keywords:** Lung cancer screening, Health disparities, Cultural adaptation, Lung cancer

## Abstract

**Background:**

Lung cancer is the leading cause of cancer-related death among Hispanic men and the second leading cause among Hispanic women in the United States. Despite evidence that lung cancer screening (LCS) reduces lung cancer mortality through early detection, Hispanic adults remain underrepresented in LCS. This study explored perceptions of LCS and shared decision-making (SDM) among Hispanic men and women to guide the cultural adaptation of the TELEhealth Shared decision-making COaching and navigation in Primary carE (TELESCOPE) intervention.

**Methods:**

Hispanic adults aged 50–77 years with a current or former smoking history of at least 20 pack-years participated in virtual focus groups. Discussions were guided by the Cultural Appropriateness Framework and explored perceptions of LCS and communication preferences. Transcripts were analyzed using rapid qualitative analysis. Two members of the research team independently reviewed transcripts, summarized findings in structured matrices, and identified themes through consensus.

**Results:**

Nineteen Hispanic adults participated in three focus groups (one in English and two in Spanish). Participants had a mean age of 60.3 years, a mean smoking history of 30.6 pack-years, 68.4% primarily spoke Spanish, and 57.8% were from South America. Four themes emerged: (1) limited awareness and understanding of LCS; (2) clear and friendly communication preferences; (3) concerns regarding fear and fatalism; and (4) importance of culturally relevant design.

**Conclusions:**

Several important themes that may influence engagement in LCS were identified. These results will inform the cultural adaptation of TELESCOPE and may guide future efforts to improve the acceptability and uptake of LCS among Hispanic adults.

## Background

Lung cancer is the leading cause of cancer-related death in the United States (U.S.) and accounts for approximately one in five cancer deaths nationally [1, 2]. Early detection through lung cancer screening (LCS) using low-dose computed tomography (LDCT) can substantially reduce lung cancer mortality, particularly when lung cancer is identified at an earlier, localized stage [3, 4]. Among Hispanic populations, lung cancer is the leading cause of cancer death among men, and the second-leading cause among women [5]. Compared with non-Hispanic White adults, Hispanic adults are less likely to be eligible for lung cancer screening (28.5% vs. 35.8% under the 2021 USPSTF criteria) [6], in part because Hispanic adults who smoke tend to accumulate fewer pack-years than White adults who smoke [7]. Among those who are eligible, they are less likely to undergo low-dose CT screening (14.23% vs. 17.69%) [8]. Moreover, in a national study of Medicare beneficiaries, estimated LCS uptake was 0.7% among Hispanic beneficiaries compared with 5.4% among White beneficiaries [9]. Hispanic individuals face several barriers to LCS, including limited awareness and knowledge of screening, language barriers, smoking-related stigma, cancer-related fear and fatalism, mistrust of the healthcare system, and limited healthcare access [10, 11]. These disparities highlight the need for culturally responsive approaches to improving LCS engagement and informed decision-making among Hispanic adults.

Evidence from the National Lung Screening Trial demonstrated that LDCT screening reduces lung cancer mortality compared with chest radiography, leading the United States Preventive Services Task Force (USPSTF) to recommend annual LDCT screening for high-risk individuals [4]. Because LCS has the potential to reduce lung cancer mortality while also involving important tradeoffs, including false positives, overdiagnosis, radiation exposure, and invasive follow-up procedures [12], the Centers for Medicare & Medicaid Services (CMS) requires a shared decision-making (SDM) visit before reimbursement for a patient’s first screening examination [13]. High-quality SDM can improve patient knowledge, reduce decisional conflict, and support informed, value-concordant screening decisions [14–16]. However, SDM conversations for LCS are often incomplete or inconsistently implemented in clinical practice, particularly in underserved populations [15, 17, 18].

Although national lung cancer screening (LCS) uptake has improved, only about one in four eligible individuals is up to date with screening, with substantial state-level variation ranging from 13 to 38% [19]. Clinician-level barriers to LCS implementation include limited familiarity with screening guidelines, insufficient time to conduct SDM discussions, competing clinical demands, and lack of support for managing screening follow-up and abnormal findings [18–20]. Patients also face barriers, including low awareness of screening, fear of cancer diagnosis, smoking-related stigma, language barriers, and limited access to care [10, 21–24]. These challenges disproportionately affect historically underserved populations, including Hispanic individuals, underscoring the need for culturally responsive approaches to LCS education and navigation.

The U.S. Department of Health and Human Services Office of Minority Health recommends culturally and linguistically appropriate interventions to improve healthcare quality, advance health equity, and reduce health disparities among diverse populations [25]. Consistent with these recommendations, growing evidence demonstrates that culturally adapted interventions improve health communication, patient engagement, acceptability of interventions, and behavioral outcomes among Hispanic populations across a variety of cancer prevention and survivorship settings [26–29]. Culturally adapted interventions for Hispanic populations have improved smoking cessation [30], cancer screening participation [31, 32], symptom management [27, 29], and patient-provider communication [28]. Adaptation frameworks emphasize modifying intervention content, language, communication, and delivery to align with the target population while preserving core evidence-based components [33, 34]. Such adaptations are particularly important in LCS because perceptions of smoking, cancer risk, and preventive care are shaped by cultural and social experiences, yet no culturally adapted LCS intervention exists for Hispanic populations. Hispanic populations are also heterogeneous with respect to language preferences, acculturation, immigration experiences, and healthcare access [35, 36], underscoring the importance of culturally responsive approaches that extend beyond direct translation alone.

To address barriers in LCS SDM and screening access, the original TELESCOPE intervention (TELEhealth Shared decision-making COaching and navigation for lung cancer screening in Primary carE) [37] was developed to deliver telehealth-based decision coaching and patient navigation to patients recruited from primary care clinics. The intervention was built on prior work demonstrating that decision coaching interventions can improve patient knowledge and decision quality regarding LCS [38, 39]. Telehealth coaching and navigation by trained patient navigators are potentially scalable and efficient strategies to support SDM and facilitate guideline-concordant screening participation among underserved populations.

## Methods

### Study design and population

This qualitative study aimed to inform the cultural adaptation of the original TELESCOPE intervention (TELEhealth Shared decision-making COaching and navigation for lung cancer screening in Primary carE) [37]. Participants took part in English- or Spanish-language focus group sessions (conducted by ACI) guided by four domains of the Cultural Appropriateness Framework (Table 1) [33], which examines how cultural values, beliefs, and preferences can inform the adaptation or development of health interventions.

**Table 1:** Cultural appropriateness framework domains and examples questions of the focus group guide

Domains	Domain description	Example focus group question
Peripheral	Programs or materials should be designed in ways that appeal to a given group (e.g., dances, songs, images)	Tell me what changes you would make to the appearance of the materials
Evidential	Sharing health information to help participants understand why a particular health issue is important	Tell me whether you would want the navigator to share statistics about lung cancer and Hispanic communities
Linguistic	Deliver programs and materials in the primary or native language of the intended group	Tell me whether there are any English or Spanish wording changes that would improve the linguistic or translation appropriateness of these materials for Hispanic patients
Sociocultural	Infusion of cultural needs, beliefs, and preferences into study design and materials to convey salience to the intended population	Tell me whether you think there are ways to better reflect Hispanic vaues like personalismo (kindness and respect), familismo (importance of family), simpatía (being polite and caring), or spirituality in our materials?

The study was designed to conduct three focus groups with approximately 6–8 participants per group, a sample size considered sufficient to capture a range of perspectives across the predefined domains of the Cultural Appropriateness Framework while remaining appropriate for in-depth focus group discussions. Potential participants were identified from the electronic health records of Hispanic primary care patients seen over the past 5 years at the Robert Wood Johnson Barnabus Health System in New Jersey. Recruitment messages were sent to 9,675 potentially eligible Hispanic patients via the MyChart patient portal. Eligibility criteria included: (1) self-identified as Hispanic and/or Latino(a), (2) able to speak and read in English and/or Spanish, (3) be 50–77 years old, (4) be a current or former smoker, and (5) have at least a 20 pack-year smoking history. Recruitment took place between October 7, 2025, and October 22, 2025. This study was approved by the Rutgers University Institutional Review Board.

### Study procedures and data collection

Prior to participation, trained bilingual study staff reviewed the study purpose, procedures, risks, and benefits with each participant by telephone and obtained verbal informed consent, which was documented in REDCap. Demographic information was collected by telephone before the focus groups using a standardized questionnaire that included age, sex, gender, race, Hispanic origin, generation, marital status, educational attainment, preferred language, and employment status. The same trained bilingual moderator conducted all English- and Spanish-language focus groups remotely via Zoom using a semi-structured interview guide. Each session lasted approximately 45 min and was audio-recorded with participants' permission. Following each focus group, participants received a $50 gift card.

### Analysis

Descriptive statistics were used to summarize participant demographics and characteristics. Qualitative data were analyzed using rapid qualitative analysis (RQA), an efficient and rigorous approach for identifying themes from interview data [40]. RQA procedures were conducted in accordance with the Planning for and Assessing Rigor in Rapid Qualitative Analysis (PARRQA) framework [40]. RQA was selected because the study had a focused objective of generating findings to inform the cultural adaptation of the TELESCOPE intervention and used a semi-structured interview guide organized around predefined domains of the Cultural Appropriateness Framework.

Spanish-language focus groups were transcribed in Spanish and subsequently translated into English. All qualitative analyses were conducted using English-language transcripts. Two members of the research team (MM and AVR) reviewed the focus group transcripts and extracted participant quotations into a structured Excel template. Quotations relevant to each interview question were organized according to predefined domains corresponding to central topics in the interview guide and summarized at the participant level. Both analysts used the same structured template, with responsibility for completing specific domains divided between MM and AVR. The predefined domains provided a deductive structure for organizing the data. MM has over 7 years of experience conducting and analyzing qualitative research, including focus groups. AVR was a Master of Public Health (MPH) student who received training and mentorship in qualitative analysis from MM for this analysis. MM and AVR completed the structured templates for their assigned domains. Within each domain, participant-level summaries and their corresponding quotations were compared to identify recurring patterns, similarities, and differences across participants and focus groups. These patterns informed the development of themes within the predefined domains. MM subsequently reviewed AVR’s completed analysis as part of quality assurance.

Trustworthiness was enhanced through structured analyst training, ongoing feedback, and quality assurance review. AVR received training and mentorship in qualitative analysis from MM, with additional oversight and feedback from JF. Training included weekly review and feedback on identifying relevant quotations, developing participant-level summaries, and identifying and developing themes. ACI, the focus group moderator, and EAC, the study PI, are bilingual Hispanic women of color; AVR is a first-generation Hispanic MPH student; and MM and JF are women of color. The team recognized that their backgrounds and experiences could influence data collection and interpretation and used independent analysis and team discussion to consider differing perspectives.

Following analysis of the three focus groups, the research team determined that the data provided sufficient depth and variation across the predefined framework domains to address the study aim and inform intervention adaptation.

## Results

A total of 180 patients expressed interest through MyChart, triggering notification to the study team for follow-up. Of these, 64 (35.5%) patients were successfully contacted by telephone, 43 (67.2%) were screened for eligibility, and 21 (32.8%) met eligibility criteria and provided informed consent. Nineteen Hispanic individuals participated in 3 focus group sessions (1 in English [*n*=6] and 2 in Spanish [*n*=6 in one FG; *n*=7 in the other FG]). The mean age was 60.3 years (SD=5.1; Table 2), and the average smoking pack-year history was 30.6 (SD=14.6). Over half were women (63.1%), over half were married (63.1%), over half (68.4%) primarily spoke Spanish, and most were from South America (57.8%).

**Table 2:** Participant characteristics (*n*=19)

Age (years, standard deviation)	60.3 (5.1)
Sex Women Men	12 (63.1%) 7 (36.9%)
Smoking pack-year history	30.6 (14.6)
Education level 8th grade or less Technical school or associate’s degree Bachelor’s or graduate degree	5 (26.3%) 9 (47.4%) 5 (26.3%)
Marital status Married Not married	12 (63.1%) 7 (36.9%)
Employment Employed full-time/self-employed Unemployed/retired Employed part-time Disabled	12 (63.1%) 5 (26.3%) 1 (5.3%) 1 (5.3%)
Race Multi-racial White Black	9 (47.4%) 9 (47.4%) 1 (5.2%)
Primary language Spanish English	13 (68.4%) 6 (21.6%)
Hispanic origin South America Caribbean Mexico	11 (57.8%) 7 (36.8%) 1 (5.4%)
Generation living in the U.S.^a^ 1 st generation 2nd generation	15 (78.9%) 4 (21.1%)

^a^ "1st generation" refers to people who were born in their native country and emigrated to one of the 50 USA states. The "2nd generation" refers to the US-born children of 1 st generation immigrants

## Themes

Below, we describe the four themes that emerged and present exemplary quotes in Table 3.

**Table 3:** Exemplary patient quotations

	Quote
1. Participants reported limited exposure to and awareness of lung cancer screening (LCS)	
1.1 LCS disadvantages not discussed	“Nobody has ever told me about the cons [of lung cancer screening].” (English-speaking participant) “I did not know there were any cons.” (English-speaking participant) “[My doctor and I discussed] just the pros, not really the cons.” (English-speaking participant)
1.2 LCS not discussed or referred	“No one has ever talked to me about that about how to prevent it [LCS]. They did a lung test because I was having trouble breathing, and that was the only time they did an exam. But they never said, “We’re going to keep monitoring this.” (Spanish-speaking participant) "My doctor never talked to me about the lung cancer screening test. I’m Colombian, but I lived in Panama for many years, and there, since I smoked, no doctor ever told me, “Go get tested,” nothing — there wasn’t good information there either. When I got here [U.S.], no one told me to get tested either. But on my own, through the internet and doing research, started looking into it and began getting the test every year. I got the tests myself. " (Spanish-speaking participant) "No, they [doctors] never asked me or told me about any test like that [LCS]. I only visit my general doctor once a year for a check-up. They check my lungs and examine me, but they never sent me anywhere to get any kind of test.” (Spanish-speaking participant)
1.3 Confusion regarding screening eligibility and potential harms	“My question is—until when am I no longer eligible?” (English-speaking participant) “What does false alarms mean? About what?” (Spanish-speaking participant) “’Lung cancer screening may not be right for someone who is not healthy.’ What does that mean? ‘Not healthy’ is what sticks to my mind.” (Spanish-speaking participant)
2. Participants preferred clear, friendly, and nonjudgmental communication for LCS shared decision-making	
2.1 Preference for clear and understandable communication	“It [incidental findings] sounds very complicated and it needs to be something everyone can understand.” (Spanish-speaking participant) “Very few people are going to understand the word ‘overdiagnosis’” (Spanish-speaking participant) “For me, I liked how you approached me personally; you were friendly, confident… You were direct, we spoke normally… That worked well for me, with trust (Spanish-speaking participant)
3. Cancer-related fear, fatalism, and appropriate risk communication influence LCS decisions	
3.1 Preference for risk communication that minimizes fear	“Now you gave me something else to be scared of. We [Hispanics] don’t like numbers. If you can eliminate those numbers [survival statistics] and just make it a friendly conversation.” (English-speaking participant) “Even though most people won’t need additional testing, don’t say ‘don’t worry’ because we will worry. We are worrisome folk.” (English-speaking participant) "Maybe if they [doctors] had told me that [to get LCS], I would’ve done it, and maybe I would have felt that same fear—the fear of hearing that I had smoking-related cancer—and maybe that would have made me quit smoking right then… And if it weren’t for me asking for it, the cancer would have spread, and maybe today I would be dead or on an oxygen tank or who knows what would have happened… It’s scary, but you have to make that decision.” (Spanish-speaking participant)
4. Culturally relevant visual design is important for LCS educational materials	
4.1 Preference for bright colors associated with Hispanic culture	“We like color. We [Hispanics] are very colorful.” (Spanish-speaking participant) “Use colors to add a little more pop… even a little red or blue, or maybe even some green… If you're sharing something with someone that you know as Latino, you want to make them feel with the colors like it's something familiar to them.” (English-speaking participant)
4.2 Preference for Latino representation in images	“Include a Latino doctor or Latino patient in pictures.” (Spanish-speaking participant) “Showing Hispanic patients would make us feel like the study is truly meant for us.” (Spanish-speaking participant)
4.3 Use realistic images	“Remove shapes/images that are not realistic. Remember, we are talking about cancer, not something minor like a cold.” (Spanish-speaking participant) “Real people would be better. The drawings don’t feel very personal.” (Spanish-speaking participant)

### Theme 1. Participants reported limited exposure to and awareness of lung cancer screening (LCS)

Participants demonstrated limited awareness and understanding of lung cancer screening (LCS), including its availability, eligibility criteria, benefits, and potential harms. Many participants reported that healthcare providers had never discussed LCS with them despite their smoking histories, and several indicated they learned about screening through personal online research rather than through clinician recommendations (Quotations 1.2). Participants noted that conversations with healthcare providers focused on the benefits of screening while providing little or no information regarding potential disadvantages (Quotations 1.1). As a result, many participants were unaware that screening could be associated with harms such as false-positive results, incidental findings, or overdiagnosis (Quotations 1.1 and 1.3).

“My doctor never talked to me about lung cancer or the lung cancer screening test.” (Spanish-speaking participant).

Participants also expressed confusion regarding screening eligibility criteria and several concepts commonly discussed during shared decision-making conversations (Quotations 1.3). Questions arose regarding how long individuals remain eligible for screening. Participants indicated that these concepts were difficult to understand and emphasized the need for clear explanations using plain language (Quotations 1.3). Collectively, these findings suggest substantial gaps in LCS knowledge and understanding among Hispanic adults and highlight the importance of educational approaches that clearly explain screening eligibility, benefits, and harms.

“I see it as saying, you don’t have money.” (Spanish-speaking participant, interpreting “until you are no longer eligible”).

“So if I was the patient, my first question would be, what do you mean the five-year survival? What does that mean?” (English-speaking participant).

### Theme 2. Participants preferred clear and friendly communication for LCS shared decision-making

Participants emphasized the importance of communication that was clear, understandable, direct, and culturally appropriate. Many participants expressed concerns about the use of complex medical terminology and indicated that information related to LCS should be presented in plain language that is easy for Hispanic individuals with varying levels of health literacy to understand (Quotations 2.1). Participants specifically noted that technical terms such as overdiagnosis and incidental findings may be unfamiliar or confusing, potentially hindering comprehension of important screening information (Quotations 2.1).

“It [incidental findings] sounds very complicated, and it needs to be something everyone can understand.” (Spanish-speaking participant).

In addition to clarity, participants emphasized the importance of a communication style that was friendly, respectful, and nonjudgmental (Quotations 2.1). Several participants described direct communication as more engaging and effective than lengthy or overly formal explanations. Participants also suggested that concise, conversational communication may help establish trust and increase receptivity to discussions about LCS and participation in related healthcare programs (Quotations 2.1).

“I think ‘direct’ is the best That’s important because when they start talking too much, people start distrusting.” (Spanish-speaking participant).

“I want to have a face-to-face with my practitioner telling me what my options are. And at that point, you give me the power to make the decision.” (English-speaking participant).

### Theme 3. Cancer-related fear, fatalism, and appropriate risk communication influence LCS decisions

Participants expressed concerns that certain approaches to communicating cancer risk could evoke fear, anxiety, and fatalistic beliefs. Several participants noted that extensive use of statistics or strongly worded risk messages could be overwhelming and potentially discourage engagement with screening (Quotations 3.1). Participants generally preferred balanced communication that acknowledged risks, provided reassurance, and emphasized the potential benefits of early detection (Quotations 3.1).

“But in my [Latin American] country, you tell somebody they got a 90 percent chance of living, they’re hearing 10 percent chance of dying.” (English-speaking participant).

Fear of receiving a cancer diagnosis emerged as an important consideration when discussing screening participation (Quotations 3.1). Participants described how concerns about learning they might have cancer could influence screening decisions and recognized that fear may prevent some individuals from seeking screening altogether. At the same time, several participants reflected on the importance of confronting these fears and emphasized that screening can enable earlier detection and treatment (Quotations 3.1).

“Maybe if they [doctors] had told me that [to get LCS], I would’ve done it, and maybe I would have felt that same fear—the fear of hearing that I had smoking-related cancer… It’s scary, but you have to make that decision.” (Spanish-speaking participant).

“Some people try to avoid reality… If there’s something preventive, well, let’s do it. Because if I get the exam and they find it in time, perfect.” (Spanish-speaking participant).

### Theme 4. Culturally relevant visual design is important for LCS educational materials

Participants emphasized the importance of culturally relevant visual design features that reflected Hispanic culture and identity. Many participants preferred bright, vibrant colors such as red and green and indicated that colorful designs were more appealing, attention-grabbing, and culturally familiar (Quotations 4.1). Participants noted that visually engaging materials would be more likely to attract attention and encourage engagement with screening information (Quotations 4.1).

“We really like color. Color is we are very colorful.” (Spanish-speaking participant).

Participants also emphasized the importance of Hispanic representation within LCS materials and expressed a preference for images depicting Hispanic patients, families, and healthcare professionals (Quotations 4.2). Several participants noted that seeing individuals who looked like them would make the materials feel more relevant and personally meaningful. In addition, participants generally preferred realistic photographs over cartoon-like images or abstract graphics, viewing realistic imagery as more appropriate and credible for communicating information about a serious topic such as cancer (Quotations 4.3). Together, these findings suggest that visual design elements may play an important role in increasing the cultural relevance and acceptability of LCS educational materials among Hispanic adults.

**“**The people in the pictures don’t look Hispanic. To me, they don’t represent us. I would identify more if I saw people who look like us.” (Spanish-speaking participant).

## Discussion

This qualitative study explored perceptions of lung cancer screening (LCS) and shared decision-making (SDM) among Hispanic adults to inform the cultural adaptation of the TELESCOPE intervention. Four themes emerged: (1) limited awareness and understanding of LCS; (2) preferences for appropriate communication; (3) concerns about fear and risk communication; and (4) the importance of visually relevant cultural designs. Collectively, these findings highlight important patient-level factors that may influence engagement in LCS and provide insight into how SDM and navigation interventions can be tailored to better meet the needs of Hispanic populations.

Our findings both aligned with and extended the four domains of the Cultural Appropriateness Framework. Preferences for culturally relevant colors, realistic images, and Hispanic representation aligned with the Peripheral domain, while preferences for understandable English and Spanish wording reflected the Linguistic domain. Participants’ responses to statistics and other risk information aligned with the Evidential domain, but further demonstrated that how such information is framed may influence fear and fatalistic beliefs about cancer. Preferences for warm, respectful, and nonjudgmental communication reflected the Sociocultural domain. Importantly, the themes did not map exclusively onto individual framework domains. Limited awareness of LCS and confusion about eligibility, benefits, and harms emerged as broader informational needs, while participants’ communication preferences intersected the Linguistic, Evidential, and Sociocultural domains. These findings suggest that culturally adapting LCS interventions may require attention to interactions across framework domains in addition to adaptations within individual domains.

Our findings are consistent with prior research demonstrating the limited reach of LCS information and educational resources among Hispanic populations, which contributes to low awareness of LCS and uncertainty about screening eligibility [10, 23, 24]. They also align with the literature highlighting the importance of culturally responsive communication and the influence of fear and fatalism on cancer prevention behaviors in Hispanic adults [33, 41, 42].Moreover, our findings extend this literature by demonstrating that Hispanic adults preferred conversational, respectful, and nonjudgmental communication; balanced discussions of screening benefits and harms that minimize fear and fatalism; and culturally relevant visual designs that reflect Hispanic communities. These findings provide specific guidance for adapting LCS decision-support interventions beyond simple language translation.

A prominent finding was participants’ limited awareness of LCS and uncertainty regarding screening eligibility, benefits, and potential harms. Many participants reported that screening had never been discussed by a healthcare provider, despite having smoking histories that may place them at elevated risk for lung cancer. However, because these findings were based on participant recall, we cannot determine whether LCS discussions did not occur or occurred but were not sufficiently salient to be recalled. Future research that incorporates healthcare providers’ perspectives and documents LCS discussions in the medical record could help distinguish among these possibilities. Participants also demonstrated gaps in understanding regarding eligibility criteria, overdiagnosis, and the purpose of screening among asymptomatic individuals. These findings are consistent with prior research demonstrating low awareness of LCS and persistent disparities in screening uptake among Hispanic populations [10, 24, 43]. These findings suggest that educational interventions targeting Hispanic adults should address not only knowledge deficits regarding LCS eligibility and purpose but also communication preferences that influence trust and engagement with screening.

Participants emphasized that culturally responsive communication extends beyond direct translation and requires attention to tone, literacy, and interpersonal style. These findings reinforce existing evidence that culturally adapted interventions should address linguistic nuances, literacy demands, and communication preferences [27, 28, 33]. Participants’ preference for warm, friendly, and trustworthy interactions is also consistent with the Hispanic cultural values of *personalismo* and *simpatía*, which emphasize the importance of personal relationships, trust, respect, and positive interpersonal interactions in health encounters [44–46]. Importantly, these interpersonal qualities may remain relevant even when providers and patients do not share a language. Empathic communication can be conveyed through nonverbal behaviors, such as welcoming body language, attentive listening, eye contact, and an unhurried manner, while trained interpreters or bilingual navigators can facilitate linguistic communication. Thus, culturally responsive LCS communication may depend on both the message—clear, nonjudgmental, and culturally appropriate information—and the messenger's ability to convey empathy, respect, and trust.

Together, these findings suggest that culturally adapted SDM interventions should attend not only to language but also to communication style and relationship-building.

Risk communication emerged as another important theme. Participants expressed concern that certain statistics, images, and phrases could evoke fear, anxiety, or fatalistic beliefs about cancer. Many participants preferred messaging that acknowledged potential harms of screening while emphasizing prevention, early detection, and opportunities for action. Participants also reacted negatively to language perceived as judgmental, coercive, or directive and instead favored communication that respected individual choice and encouraged dialogue. These findings suggest that SDM tools for Hispanic adults should frame screening discussions in ways that promote autonomy while minimizing fear, stigma, and perceptions of judgment.

Participants also emphasized the importance of culturally relevant visual design, including the use of familiar colors such as red or green, realistic imagery, and Hispanic representation [28, 33, 47]. Although visual adaptations are often considered peripheral, participants viewed these elements as important signals that educational materials were intended specifically for Hispanic communities. These findings suggest that culturally relevant visual design may enhance the perceived relevance, trustworthiness, and acceptability of LCS educational materials.

These findings have important implications for LCS shared decision-making. Our results suggest that effective SDM for Hispanic adults should extend beyond communicating screening eligibility and risks to incorporate culturally responsive messaging, attention to fear and fatalism, and educational materials that are linguistically and culturally relevant. Collectively, these findings provide practical guidance for the cultural adaptation of LCS decision-support and navigation interventions for Hispanic populations.

Several limitations should be considered when interpreting these findings. Participants were recruited from a single academic center in New Jersey, which may limit the generalizability to Hispanic populations in other geographic regions and healthcare settings. Additionally, because multiple participants spoke in a group setting, the audio recordings did not always allow individual speakers to be reliably distinguished during transcription. Consequently, unique participant IDs could not be consistently assigned to quotations, limiting our ability to assess their distribution across participants. In addition, participants were identified through the health system’s electronic health records, which may have limited representation of individuals who are less engaged with the healthcare system. Because focus groups were conducted virtually, the study may also have underrepresented individuals with limited access to or comfort with digital technology. These groups may experience additional barriers to LCS engagement that were not fully captured in our findings. Moreover, we did not collect information on prior LCS or lung cancer history, which may have influenced participants’ perspectives on screening. Nevertheless, our study included participants representing diverse Hispanic backgrounds, countries of origin, language preferences, and levels of acculturation, providing a broad range of perspectives. Findings highlight important cultural, linguistic, and communication factors that may influence engagement in LCS among Hispanic adults. Future research should evaluate whether incorporating these preferences into culturally adapted navigation and decision-support interventions improves SDM quality, intervention acceptability, and ultimately LCS uptake among Hispanic adults.

## Data Availability

Deidentified data from this study are not available in a public archive but can be made available by emailing the corresponding author.
